# A Retrospective Study on Patellar Desmopathy Following Surgical Resolution of Cranial Cruciate Ligament Rupture in 28 Dogs

**DOI:** 10.3390/ani15071052

**Published:** 2025-04-04

**Authors:** Francisco Vidal-Negreira, Victoria Valiño-Cultelli, Mario García-González, Óscar Varela-López, Jose-Daniel Barreiro-Vázquez, Antonio González-Cantalapiedra

**Affiliations:** 1 Rof-Codina Veterinary Teaching Hospital, Faculty of Veterinary, Universidad de Santiago de Compostela, 27002 Lugo, Spain; oscar.varela@usc.es (Ó.V.-L.); antonio.cantalapiedra@usc.es (A.G.-C.); 2 Department of Anatomy, Animal Production and Veterinary Clinical Sciences, Veterinary Faculty, Universidad de Santiago de Compostela, 27002 Lugo, Spain; victoria.cultelli@usc.es (V.V.-C.); mariog.gonzalez@usc.es (M.G.-G.); josedaniel.barreiro@usc.es (J.-D.B.-V.); 3 3B’s Research Group, I3Bs—Research Institute on Biomaterials, Biodegradables and Biomimetics, University of Minho, Headquarters of the European Institute of Excellence on Tissue Engineering and Regenerative Medicine, Barco, 4805-017 Guimarães, Portugal; 4 ICVS/3B’s—PT Government Associate Laboratory, Barco, 4805-017 Guimarães, Portugal

**Keywords:** desmopathy, patellar ligament, TPLO, cranial cruciate ligament, lameness, radiography, dog

## Abstract

Cranial cruciate ligament rupture is the most common orthopedic condition in dogs, causing lameness, joint instability, and discomfort due to its role in knee stability. Surgical treatment, particularly osteotomies in the proximal tibia, is the preferred approach, with tibial tuberosity advancement (TTA), tibial plateau leveling osteotomy (TPLO), and the modified Maquet technique (MMT) being widely used. A common postoperative complication is patellar desmopathy—thickening of the ligament that is detectable on radiographs and can cause further discomfort. However, studies on this complication in TPLO are limited, and there are no direct comparisons of ligament thickening between these surgical techniques. This study aimed to determine the prevalence of ligament desmopathy in TPLO, examine any associations with age, sex, or weight, and compare findings with those from other techniques. A retrospective analysis was conducted, with ligament thickness measured at three points during postoperative follow-up, which had a mean of approximately 2.5 months. Results showed an 86.7% prevalence of desmopathy, with average thicknesses of 3.90 mm proximally, 4.69 mm at mid-ligament, and 5.98 mm distally. These findings align with previous TPLO studies and suggest that TPLO may cause greater ligament thickening than TTA or MMT, particularly in the distal portion.

## Simple Summary

Cranial cruciate ligament rupture is one of the most common orthopedic issues in dogs, often causing pain, limping, and instability in the knee joint. Surgical procedures are usually required to restore stability. A common complication following these surgeries is patellar desmopathy, which can cause additional discomfort and affect postsurgical recovery. This study aimed to determine how often this complication occurs after TPLO and whether factors such as age, weight, or sex of the dog influence the likelihood of this complication. We found that desmopathy affected most dogs within two and a half months after surgery, especially in the distal part of the ligament. Our findings suggest that TPLO may lead to more ligament thickening than other methods. These results can help veterinarians make informed decisions about treatment options and highlight the importance of monitoring ligament health during recovery to ensure a better outcome regarding mobility and comfort.

## 1. Introduction

Cranial cruciate ligament rupture (CCLR) is one of the most frequently diagnosed orthopedic conditions in dogs, leading to pain, lameness, and instability in the knee joint [1,2,3]. This condition severely impacts the dog’s mobility and quality of life, and surgical intervention is often necessary to restore stability [4,5,6]. The most used surgical techniques are tibial osteotomies, including tibial plateau leveling osteotomy (TPLO) [7], tibial tuberosity advancement (TTA) [8], and the modified Maquet technique (MMT) [9]. These procedures have shown favorable outcomes in terms of joint stabilization and long-term recovery [10,11]; however, like any surgical intervention, they carry the risk of complications. One such complication is patellar ligament desmopathy [12,13], a condition defined by thickening of the patellar ligament, often associated with inflammatory responses in the ligament tissue [14].

Patellar ligament desmopathy, while not as immediately severe as other surgical complications, nonetheless, can affect the postoperative recovery and long-term comfort of the animal [15]. This ligament thickening can be accompanied by pain, reduced joint function, and lameness [14], potentially compromising the surgical outcome. Although the precise etiology of this condition remains uncertain, inflammatory reactions caused by surgical manipulation, intraoperative trauma, increased tension in the ligament following TPLO, or excessive postoperative exercise are commonly proposed factors [16,17]. Infectious causes are considered rare due to the limited vascularization provided to the ligament, leading researchers to focus on inflammation as the main underlying mechanism [18].

The incidence of patellar ligament desmopathy following TPLO has been reported to range between 1% and 5% in previous studies [19,20], but there is limited data on the factors that may influence its occurrence or severity [14,15]. Radiographic analysis remains the most commonly used imaging modality in both clinical and research contexts for diagnosing this condition, typically based on increased ligament thickness in postoperative follow-up compared to baseline values [15]. Although other imaging techniques, such as ultrasonography or MRI, may offer greater detail, radiography remains widely used due to its availability, objectivity, and cost-effectiveness in clinical follow-up evaluations [21].

Despite its clinical relevance, few studies have investigated patellar ligament desmopathy specifically in the context of TPLO, and, to date, there have been no direct comparisons of ligament thickening among TPLO, TTA, and MMT. Understanding the factors that contribute to ligament desmopathy is critical for refining surgical approaches and improving patient outcomes. This study aims to address this gap by assessing the prevalence of patellar ligament desmopathy in dogs following TPLO, analyzing potential associations with variables such as age, sex, and weight. Through this analysis, we hope to determine whether TPLO poses a higher risk for ligament thickening than alternative techniques and provide insights that could guide clinical decision-making and postoperative care.

## 2. Materials and Methods

This retrospective study was conducted in accordance with the European Directive 2010/63/EU of 22 September 2010 on the protection of animals used for scientific purposes. It was also approved by the Ethics Committee of the Galician Public Foundation Rof Codina (Lugo) (AELU001/21/INVMED(2)/ANIMAL(05)/AGC/01).

### 2.1. Patient Selection

The patient selection was based on the database of the Rof Codina University Veterinary Hospital (Lugo, Spain). The search was filtered to identify dogs diagnosed with CCLR between 1 May 2022 and 31 January 2024 who had been treated with TPLO. The terms used in the database included “TPLO”, “lameness”, “cranial cruciate ligament rupture”, and “CCLR”.

The inclusion criteria for the study were as follows: skeletally mature dogs, diagnosis of cranial cruciate ligament rupture, treatment of the rupture with TPLO performed by an experienced surgeon, and availability of preoperative and postoperative control radiographs.

The exclusion criteria included other surgical complications, other orthopedic issues in the affected limb aside from the cranial cruciate ligament rupture, the presence of metabolic pathologies, poor radiographic technique hindering proper evaluation of the patellar ligament, or any deviation from the specified anesthetic or surgical protocol.

For patients meeting these criteria, the following data were collected: name and medical record number, breed, age at the time of surgery, sex, weight, general physical and orthopedic examination findings, affected limb, surgical and anesthetic protocol, discharge instructions, and preoperative and postoperative radiographs in laterolateral and craniocaudal views.

### 2.2. Anesthetic and Surgical Protocol

In this retrospective study, it was verified through hospital records that all TPLO surgeries were performed following the same surgical protocol previously described by Slocum and Slocum (1993) [7] and by the same experienced surgeon. Meniscal integrity was assessed by an arthroscopic approach.

The anesthetic protocol was the same in all surgeries. Briefly, patients were premedicated with a combination of dexmedetomidine (5 μg/kg IM, Dexdomitor 0.5 mg/mL, Orion Corporation, Madrid, Spain) and morphine (0.3 mg/kg IM, Morfina B. Braun 20 mg/mL, B. Braun Medical, Barcelona, Spain). Once the patient was sedated, one of the cephalic veins was catheterized (Abbocath, size adjusted to patient, ICU Medical, Spain), and fluid therapy was initiated with Lactated Ringer’s solution (5 mL/kg/h, B. Braun, Barcelona, Spain). Additionally, a dose of cefazolin (22 mg/kg IV Cefazolin Normon 1 g, Normon Laboratories, Madrid, Spain) was administered 30 min prior to surgery and 60 min after the first incision.

Propofol (2 mg/kg IV, Propovet 10 mg/mL, Zoetis Spain, Madrid, Spain) was then used for induction, followed by a continuous infusion of morphine (0.123 mg/mL IV), 5% lidocaine (1.5 mg/mL IV, Lidocaína B. Braun 5%, B. Braun Medical, Barcelona, Spain), and ketamine (0.6 mg/mL IV, Ketamidor 100 mg/mL, Wels, Austria) (M.L.K) at 1 mL/kg/h to maintain intraoperative analgesia. After orotracheal intubation, the patient was connected to an appropriate ventilation system, and the anesthetic plane was maintained using sevoflurane (SevoFlo^®^, Esteve Veterinary, Barcelona, Spain) between 1.5 and 2.5 CAM with a 0.5 L/h O_2_ flow.

At the end of the surgery, a dose of meloxicam (0.2 mg/kg IV, Meloxidolor 5 mg/mL, Le Vet Beheer, Oudewater, The Netherlands) was administered as an anti-inflammatory.

After skin suturing, a Robert-Jones bandage was applied and then removed after 24 h. All patients received the same postoperative treatment, which consisted of meloxicam 0.1 mg/kg orally once daily for seven days and cefazolin 22 mg/kg orally twice daily for ten days. Additionally, exercise restriction was advised until the follow-up radiograph at two and a half months.

Any patient who deviated from this protocol was also excluded from the study.

### 2.3. Radiographic Evaluation

All radiographs were taken with the patient under sedation (dexmedetomidine 5 µg/kg and butorphanol 0.2 mg/kg, both IM) and using the same equipment (Luminos Fusion FD, Siemens, Erlangen, Germany). For radiographic evaluation, we used the open-source DICOM image management software Horos^®^ V. 3.3.3 (Nimble Co., Annapolis, MD, USA, https://horosproject.org/ (accessed on 3 June 2024)). The following parameters were measured: preoperative and postoperative tibial plateau angle (TPA), the length of the patellar ligament, and its thickness.

**Figure 1:**
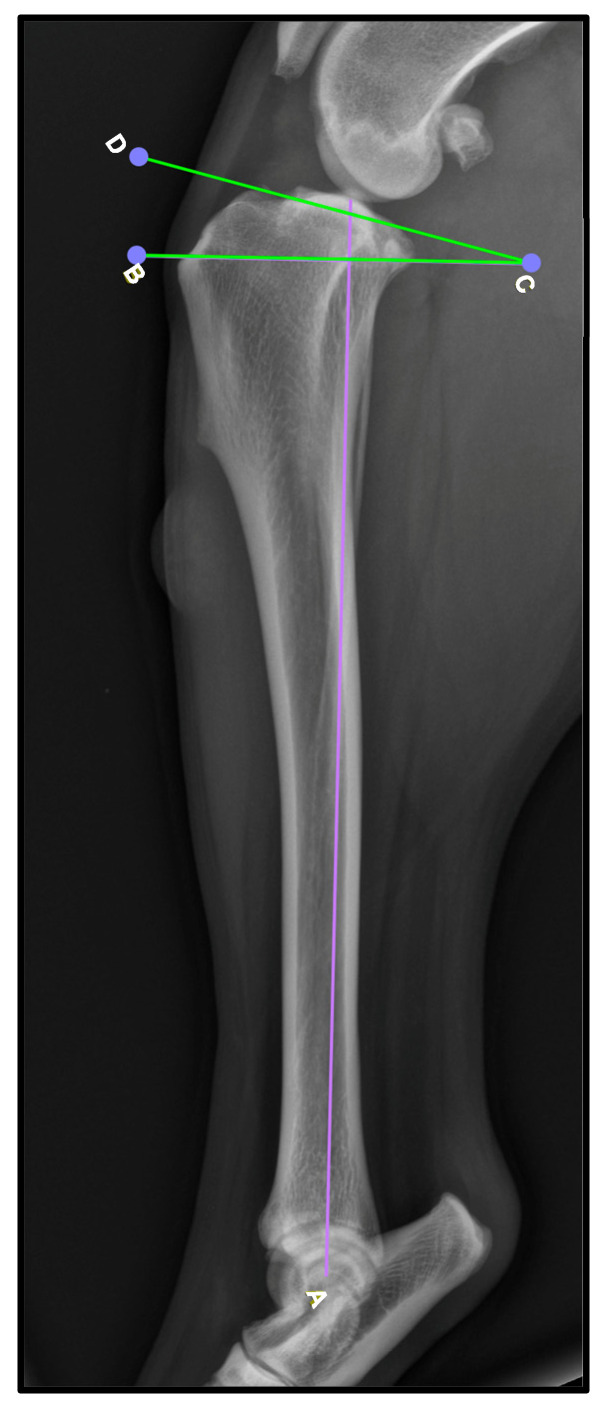
Determination of the tibial plateau angle. A: line parallel to the tibial sagittal plane; B–D: line perpendicular to the previous one. The tibial plateau angle is highlighted in green.

To obtain these measurements, the lateral view was used. To determine the TPA (Figure 1), the mechanical axis of the tibia is drawn by connecting the midpoint of the intercondylar eminence to the center of the talocrural joint [A, Figure 1]. Then, a second line is drawn along the slope of the medial tibial condyle to establish the tibial plateau line [D–C, Figure 1]. Finally, a perpendicular line to the mechanical axis is created [B–C, Figure 1], and the TPA is determined as the angle formed between this line and the tibial plateau line. While TPA was recorded, no statistical analysis was performed to assess its relationship with patellar ligament thickening, as this was not the primary focus of the study.

**Figure 2:**
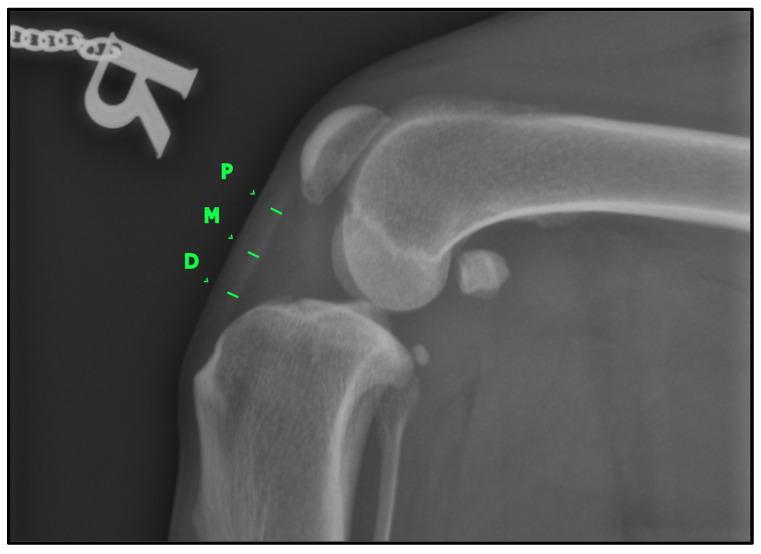
Lateral knee radiographic view (LL) illustrating the measurement of patellar ligament thickness. The ligament was divided into four equal segments, and thickness was recorded at three predefined reference points: proximal (P), middle (M), and distal (D). These measurement locations are marked in green for clarity.

To determine the thickness of the patellar ligament (Figure 2), we followed the method previously described by DeSandre-Robison et al. (2017) [14]. To standardize measurements across patients, the total length of the patellar ligament was first measured from its proximal insertion at the patella to its distal insertion at the tibial tuberosity. This total length was then divided into four equal segments. The ligament thickness was measured at the three junction points between these segments, ensuring that reference points were proportional to ligament length in each patient. Taking this into account, three points were measured in every patient: one proximal, one distal, and one in the middle of the ligament.

All three measurements were taken both in the preoperative radiograph (T-0) and in the follow-up radiograph (T-1). All radiographs were anonymized by assigning a random number to each. An increase in ligament thickness compared to the preoperative measurement was considered indicative of desmopathy, as stated in other studies [15].

### 2.4. Statistical Analysis

For the statistical analysis, we used the Sigma Plot V. 12.5 software (Systat Software Inc., San Jose, CA, USA). The variable analyzed was the ligament thickness, measured at three anatomical points (proximal, middle, and distal) both pre- (T-0) and postoperatively (T-1), as well as the difference between these values. The independent variables included sex, weight, and age at the time of surgery. Time between surgery and first follow-up was considered a covariable.

A descriptive analysis was performed, with results presented as mean ± standard deviation. Additionally, the normality of ligament thickness at the three measurement points was evaluated using the Shapiro–Wilk test. When the normality hypothesis was rejected, non-parametric methods were chosen for variable analysis.

The Kruskal–Wallis test was used to compare ligament measurements based on their anatomical location and preoperative and postoperative time points. For post hoc analysis, Tukey’s test was employed to specify which groups showed significant differences.

To examine the influence of age, patients were divided into two groups: one group (age ≤ 84 months) and a second group (age > 84 months). A similar approach was applied for sex, with one group for males and another for females, and for weight, with a first group (weight ≤ 30 kg) and a second group (weight > 30 kg).

Once the groups were established, the difference in ligament thickness for each patient was calculated at the three measurement points. Finally, the Mann–Whitney–Wilcoxon test was used to compare these differences between the described groups.

Additionally, we assessed whether the variability in follow-up time influenced ligament thickness. A Spearman correlation test was performed between the number of days from surgery to follow-up radiographs and the changes in ligament thickness at the three measurement points (proximal, middle, and distal).

For all statistical tests applied, a significance threshold of *p* < 0.05 was set.

## 3. Results

### 3.1. Descriptive Analysis

A total of 89 skeletally mature patients who underwent TPLO were initially studied, out of which 28 animals were ultimately included in this study. In two of these dogs, TPLO was performed on both limbs in separate anesthetic sessions. The remaining 61 patients were excluded due to missing radiographs or deviations from the radiographic, surgical, or anesthetic protocols.

Among the 28 dogs who completed the study, there were eight mixed breeds (28.6%), three German Shepherds (10.7%), two Labradors (7.1%), two Boxers (7.1%), two Golden Retrievers (7.1%), one American Staffordshire Terrier, one Bulldog, one Great Dane, one Miniature Pinscher, one Dogue de Bordeaux, one Bullmastiff, one Border Collie, one American Akita, one West Highland Terrier, one Mastiff, and one Can de Palleiro (each representing 3.6%).

Of these 28 animals, 8 were males (28.6%) and 20 were females (71.4%). The mean age at the time of surgery was 79.8 ± 33.6 months, with a range of 24 to 156 months. The average weight was 32.9 ± 11.3 kg (kg), with a range of 9 to 51 kg. A total of 46% of the dogs were operated on the right hind limb, 46% on the left hind limb, and 8% on both hind limbs in separate anesthetic sessions.

The interval between surgery and postoperative evaluation or follow-up was 80.3 ± 30.0 days, with a range of 30 to 189 days. The patellar ligament length was 4.4 ± 0.9 cm, ranging from 2.6 cm to 6.1 cm. The preoperative tibial plateau angle was 26.0 ± 4.1 degrees (°), while the postoperative angle was 3.1 ± 2.0°, with ranges of 18.2° to 38.4° preoperatively and 0.2° to 8.0° postoperatively.

Of all the limbs evaluated, 86.67% (24 patients) exhibited radiographic signs of patellar ligament desmopathy at the time of follow-up, while 13.3% (4 patients) showed no evidence of desmopathy.

Finally, to assess the patellar ligament thickness, the mean thickness was calculated for each measurement point, as well as for the three preoperative and postoperative measurement points. The results are presented in Table 1.

### 3.2. Statistical Analysis

**Figure 3:**
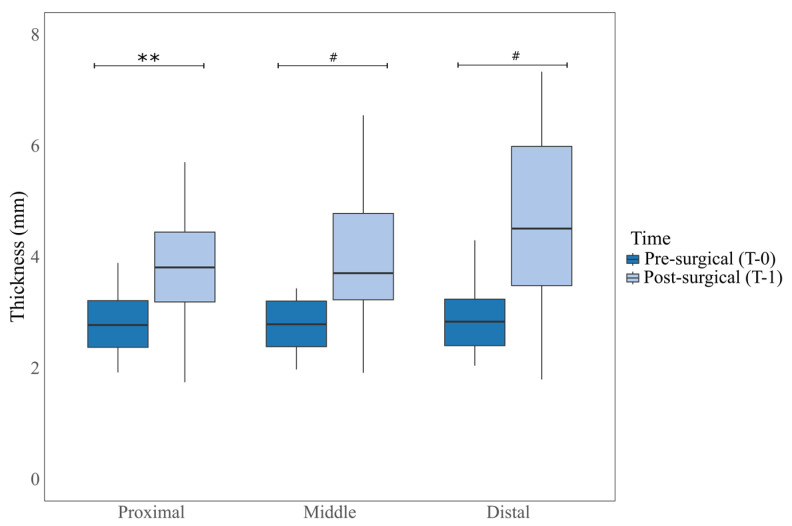
Box plot representation of measurements at different anatomical points, preoperative and postoperative. Statistically significant differences between groups at ** *p* < 0.01, ^#^ *p* < 0.001.

For the statistical analysis, preoperative and postoperative measurements at each measurement point were compared using the Kruskal–Wallis test. Statistically significant differences were observed between groups beyond what would be expected by random chance (H: 62.63, *p* < 0.001). In the post hoc tests, statistically significant differences were found at all measurement points, with the distal point (mean rank difference = 2033, *p* < 0.001), the midpoint (mean rank difference = 1839, *p* < 0.001), and the proximal point (mean rank difference = 1527, *p* = 0.002). These differences are shown in Figure 3.

Comparisons between the previously described groups for age (*p* = 0.205), sex (*p* = 0.06), and weight (*p* = 0.567) did not yield statistically significant differences in any of these variables. The mean ± standard deviation for each group is shown in Table 2.

Additionally, a Spearman correlation test was performed between follow-up time and ligament thickness differences at the three measurement points. No statistically significant correlations were found (proximal: ρ = −0.34, *p* = 0.065; middle: ρ = −0.14, *p* = 0.445; distal: ρ = −0.10, *p* = 0.581).

## 4. Discussion

The present study has demonstrated that, following TPLO surgery for the resolution of cranial cruciate ligament rupture (CCLR) in dogs, the prevalence of tibial-patellar ligament desmopathy at two and a half months post-surgery is 86.67%. This finding aligns with previous studies, which reported ligament thickening in 80–100% of patients undergoing this surgical technique within the same postoperative period [14,15,21]. The timing of the thickening also corresponds to other studies evaluating TPLO postoperative complications, where radiographic thickening of the ligament was reported to occur between the fifth- and eighth-weeks post-surgery [19,20], consistent with the timeframe observed in this study.

When compared to other studies on this condition, the thickening observed at the three measurement points during the postoperative evaluation in this study was similar to the findings reported in TPLO by DeSandre-Robinson et al. (2017) [14], with 3.54 ± 1.62 mm vs. 3.90 ± 1.12 mm at the proximal measurement and 5.48 ± 2.75 mm vs. 5.98 ± 3.78 mm at the distal measurement. However, no data were provided regarding the mid-ligament measurement [14]. In another study by Carey et al. (2005) [15], the mean values obtained were slightly higher than those reported in this study, with 5.5 ± 3.3 mm vs. 3.90 ± 1.12 mm at the proximal measurement, 6.5 ± 3.4 mm vs. 4.69 ± 2.16 mm at the mid-ligament, and 8.5 ± 3.9 mm vs. 5.98 ± 3.78 mm at the distal measurement [15]. While similar measurement protocols were used, variations in imaging techniques and anatomical landmarks across studies must be considered when making direct comparisons. Given these limitations, our study presents these comparisons descriptively in the discussion rather than integrating them into the statistical analysis.

Also, considering the preoperative and postoperative tibial plateau angles reported in previous studies, the findings of the present study show a comparable preoperative angle but a greater reduction postoperatively. Carey et al. (2005) [15] documented a preoperative tibial plateau angle of 24° ± 4.3° (range: 17–43°) and a postoperative angle of 6° ± 3.0° (range: 0–14°) [15]. Similarly, Desandre et al. (2017) reported a preoperative angle of 29.35° ± 6.33° (range: 16.0–50.0°) and a postoperative angle of 12.61° ± 4.03° (range: 0.0–20.0°) [14]. Mattern et al. (2006) [21] found a preoperative tibial plateau angle of 24.9° ± 3.61° (range: 17–31°) and a postoperative angle of 6.3° ± 2.81° (range: 2.5–12.1°). In comparison, the present study reported a preoperative tibial plateau angle of 26.0° ± 4.1° (range: 18.2–38.4°) and a postoperative angle of 3.1° ± 2.0° (range: 0.2–8.0°), suggesting a more pronounced correction of the tibial plateau slope. However, no statistical analysis was performed to assess whether this correction influenced patellar ligament thickening, as this was not the primary focus of the study.

The differences between the results obtained in this study and the previous ones could be attributed to several factors. One of them is the surgical protocol, as in Carey et al. (2005) [15], where surgeries were performed by multiple surgeons using different approaches, which sometimes included patellar luxation or intraoperative arthrotomy [15]. These variations in the surgical protocol, particularly the use of arthrotomy, may have led to greater thickening of the tibial-patellar ligament. This approach, commonly performed for meniscal exploration, involves incisions in the peripatellar area, placing tension on the ligament and potentially causing its thickening [14,22].

When comparing other techniques, DeSandre-Robinson et al. (2017) [14] also measured tibial-patellar ligament thickening after performing TTA using a similar methodology to this study. This technique yielded values of 3.20 ± 0.64 mm at the proximal measurement and 4.73 ± 2.14 mm at the distal measurement, without reporting data for the mid-ligament measurement [14]. Similarly, another study by Kühn et al. (2011) [22], also conducted in dogs after TTA, reported similar results, with 4.5 mm at the proximal measurement and 4.3 mm at the distal measurement, again without providing data for the mid-ligament measurement [22].

In another technique, MMT, Valiño-Cultelli et al. (2022) [23] reported mean thickening values of 3.31 ± 1.48 mm at the proximal measurement, 3.88 ± 1.74 mm at the mid-point, and 4.46 ± 1.83 mm at the distal measurement. These values do not differ significantly from those obtained for TTA [23]. Furthermore, in their study, it was demonstrated that ligament thickness tends to increase during the first eight weeks post-surgery, followed by a decrease between the second and fifth postoperative months [23]. All comparisons with this and other techniques are summarized in Table 3. However, these comparisons must be interpreted with caution, as variations in radiographic methodology, measurement landmarks, and patient populations among studies may influence the reported values.

With these techniques, there appears to be less thickening of the ligament in the distal measurement compared to that observed in the TPLO studies and this work. To explain this thickening, which is more pronounced in the distal portion of the ligament, Boudrieau et al. (2009) [12] proposed the lever-arm theory. In this model, one can imagine a lever inserted at the contact point between the femur and tibia, which pushes downward at the insertion point of the patellar ligament. In a normal knee, this point is located slightly distal to the center of the ligament, distributing forces evenly across it. During joint movement, a certain amount of force would be required to move the lever downward [12].

However, after TPLO, the lever arm shortens by up to 10%. This shortening increases the force required to mobilize the limb, thereby increasing the stress on the ligament. Consequently, it is also important to avoid rotating the proximal osteotomy fragment, as this would further shorten the lever arm and place additional stress on the ligament [12].

This effect is not observed in TTA because, by advancing the tibial tuberosity and thus the insertion point, the lever arm lengthens by at least 10%. This lengthening reduces the force required to mobilize the joint, resulting in less tension on the ligament [12]. This finding aligns with other studies on ligament thickening following TPLO [15,21]. However, it is important to note that this theory has not been confirmed in experimental studies, leaving it as an open field for future research [12].

These biomechanical alterations following TPLO may contribute to the increased stress observed in the tibial-patellar ligament, particularly in its distal portion. Mattern et al. (2006) [21] suggested that changes in stifle biomechanics could play a key role in the development of ligament desmopathy, as reduced tibial plateau angles postoperatively were associated with increased longitudinal and transverse thickness, as well as the cross-sectional area, of the distal portion of the ligament. Their findings indicated a trend toward significant ligament thickening when the postoperative tibial plateau angle was below 6°, reinforcing the idea that increased mechanical stress may contribute to the development of patellar ligament desmopathy following TPLO [21].

Other possible explanations for distal ligament thickening include intraoperative trauma, increased tension in the distal portion of the ligament after surgery, and excessive exercise during postoperative recovery [16,19]. While some authors have suggested that a greater reduction in TPA could contribute to ligament thickening [19], this was not evaluated in our study. Excessive exercise could not only explain localized thickening in the distal portion but also generalized thickening throughout the entire ligament [15].

Intraoperatively, the ligament may also sustain trauma during its separation using Gelpi retractors, due to proximity to the osteotomy saw, heat generated during bone cutting, or the placement of the Kirschner wire near the ligament’s insertion [15].

In this study, damage from heat or proximity to the saw is unlikely, as records indicate that the ligament was separated to prevent contact, and sterile saline was irrigated during the cut to avoid overheating. Similarly, it is improbable that the Kirschner wire caused damage to the distal portion of the ligament, as it was inserted directly into the proximal osteotomy fragment without coming near the ligament. However, this theory might explain localized thickening in the distal portion of the ligament in TTA, where the Kirschner wire is inserted near the ligament’s insertion, potentially causing trauma in the area that could result in localized and sustained postoperative thickening [22].

Other studies have shown that weight is a risk factor for greater ligament thickening [21]. This is related to the increased load borne by the limb, which generates greater tibial stress. As a result, the stress placed on the ligament during postoperative correction increases, leading to greater thickening in heavier animals [12,21]. However, other studies found no relationship between animal weight and ligament thickening [14,15]. This study reached the same conclusion, as heavier patients showed no significant differences in ligament thickening compared to lighter patients. This supports the findings of these studies, but further research is needed to confirm whether this variable influences patellar ligament thickening.

In human medicine, age has been identified as a potential risk factor for the development of patellar desmopathy, along with other causes [24]. However, there is limited evidence in veterinary medicine to suggest a direct relationship between age and patellar ligament thickening following TPLO. While older patients may experience differences in healing capacity and tissue remodeling, this study did not find a significant correlation between age and ligament thickening reporting similar findings as in other studies [15]. Further research is needed to determine whether age plays a role in postoperative ligament changes in dogs undergoing TPLO.

All patients were given the same rest instructions until the follow-up, but compliance with these instructions was not verified with the owners. Therefore, it is not possible to determine whether the observed ligament thickening was due to inadequate rest or another cause, as theorized in the study by DeSandre-Robinson et al. (2017) [14]. For future studies, it would be useful to include a questionnaire to assess the physical activity level of patients during the rest period. This would allow for a more in-depth analysis of this variable and help establish whether increased postoperative activity affects patellar ligament thickening.

For radiographic evaluation, digital images were used, allowing for adjustments in parameters such as contrast to highlight the ligament’s edges. This technique is minimally invasive and commonly used for diagnosing CCLR and postoperative follow-ups [25], as physical examination has demonstrated not being accurate enough to diagnose patellar desmitis [26]. Its use during surgical planning and postoperative evaluations facilitates both surgical follow-up and monitoring of patellar ligament thickening [14,25]. However, as described in other studies, radiography does not distinguish between ligament-related changes and those in adjacent structures [14,15]. Furthermore, radiographic interpretation of ligament edges is subjective, which could introduce measurement errors [14]. Therefore, future studies should consider supplementing ligament thickening assessments with ultrasonographic evaluations, a minimally invasive method that can more accurately differentiate between ligament-specific changes and those in surrounding tissues [14,27].

To overcome these limitations, ultrasonographic evaluations have become a well-established, minimally invasive method for assessing ligament thickening, allowing for a more accurate differentiation between ligament-specific changes and those in surrounding tissues [14,28]. Recent studies have demonstrated that sonoelastography may provide additional insights into ligament adaptation following TPLO. Signore et al. (2024) [29] found increased stiffness in the patellar ligament after TPLO and TTA, suggesting that elastographic evaluation could be useful in assessing postoperative ligament changes [29]. Additionally, Pennalisco et al. (2024) [28] highlighted that elastographic alterations in the patellar ligament may begin even before surgical intervention in dogs with cranial cruciate ligament disease [28]. These findings emphasize the potential of sonoelastography for monitoring ligament integrity both pre- and postoperatively.

Although all radiographs were taken with the joint flexed at a 135° angle, minor variations were observed between radiographs and patients. While angular variations did not show statistically significant differences in ligament thickness in other studies evaluating desmopathy post-TTA [30], no studies have assessed whether such variations affect measurements in TPLO patients. Future studies should account for radiographic angles and determine whether small joint angle differences significantly impact radiographic measurements of patellar ligament thickness.

Another limitation of this study, due to its retrospective and clinical nature, is the variability in follow-up times. The interval between surgery and the first postoperative evaluation ranged from 30 to 189 days, with a mean follow-up time of 80.3 ± 30.0 days. This variability may have contributed to differences in ligament thicknesses between patients, as seen in studies by Carey et al. (2005) [15], DeSandre-Robinson et al. (2017) [14], and Valiño-Cultelli et al. (2022) [23]. However, in the present study, no significant correlation was found between follow-up time and ligament thickness at any of the three measurement points (proximal: ρ = −0.34, *p* = 0.065; middle: ρ = −0.14, *p* = 0.445; distal: ρ = −0.10, *p* = 0.581). These findings suggest that, despite the variability in follow-up times, this factor did not introduce a systematic bias in the results. Moreover, as previously mentioned, studies on TTA and MMT have shown an initial increase in ligament thickness that decreases after eight weeks post-surgery [15,21,22]. This trend was not observed in studies evaluating thickening after TPLO [14,15,21]. Future studies with standardized follow-up intervals could further explore the potential impact of follow-up timing on ligament thickening.

Finally, due to the retrospective nature of the study and to ensure animal welfare, NSAIDs were used to manage postoperative pain and inflammation. These may have influenced ligament thickening during the early postoperative period [31], though no studies in dogs have demonstrated this. The administration of NSAIDs for treating ligament lesions is diverse. While some studies demonstrated that the administration of NSAIDs reduced the inflammation of the patellar ligament in rats [32] and humans [33], others differ, arguing that the NSAIDs may impair ligament healing [34,35]. Additionally, surgical interventions like TPLO have been shown to decrease blood delivery to the patellar tendon, potentially influencing the NSAIDs’ effect on its thickening [36]. Future studies should investigate whether the administration of these drugs affects patellar ligament thickening.

## 5. Conclusions

Desmopathy is a common finding two and a half months after TPLO, with a prevalence of 86.7% observed at the postoperative follow-up, particularly pronounced in the distal portion of the ligament. Interestingly, this study found no significant influence of sex, weight, or age on tibial-patellar ligament thickening. Furthermore, the thickening associated with TPLO was more pronounced than that reported for TTA or MMT in comparative studies, suggesting a potential clinical advantage for TTA. However, additional clinical research directly comparing these techniques is essential to validate these observations and refine the understanding of their outcomes.

## Figures and Tables

**Table 1:** Mean ± standard deviation of ligament thickness, in millimeters, at pre-surgical and post-surgical measurement points.

	Pre-Surgical (T-0)			Post-Surgical (T-1)		
	Proximal	Middle	Distal	Proximal	Middle	Distal
	2.81 ± 0.54	2.81 ± 0.6	2.93 ± 0.65	3.90 ± 1.12	4.69 ± 2.16	5.98 ± 3.78
Mean	2.85 ± 0.52			4.86 ± 2.20		

**Table 2:** Mean ± standard deviation of the difference in ligament thickness, in millimeters, for each group of age, weight, and sex, and for each measure point.

	Group	Measurement Point	Mean ± Standard Deviation
Age	≤84 months	Proximal	1.214 ± 0.911
		Middle	2.119 ± 2.074
		Distal	3.657 ± 3.570
	>84 months	Proximal	0.952 ± 0.813
		Middle	1.634 ± 1.307
		Distal	2.444 ± 1.505
Sex	Male	Proximal	0.923 ± 0.862
		Middle	1.373 ± 1.178
		Distal	2.125 ± 1.470
	Female	Proximal	1.404 ± 0.850
		Middle	2.882 ± 2.745
		Distal	4.903 ± 3.124
Weight	≤30 kg	Proximal	0.786 ± 0.612
		Middle	1.614 ± 1.457
		Distal	2.441 ± 1.826
	>30 kg	Proximal	1.343 ± 1.044
		Middle	2.106 ± 1.448
		Distal	3.584 ± 2.398

**Table 3:** Summary of results (mean ± standard deviation) from this study and the studies on TPLO, TTA, and MMT that were mentioned. When information for a specific measurement point was not provided, it is represented as “-”. It can be observed that TPLO studies report greater ligament thickness compared to TTA or MMT studies. Source: own elaboration [14,15,22,23].

Technique	Study	Ligament Thickness (mm)		
		Proximal	Middle	Distal
TPLO	This study	3.90 ± 1.12	4.69 ± 2.16	5.98 ± 3.78
	Carey et al., 2005 [15]	5.5 ± 3.3	6.5 ± 3.4	8.5 ± 3.9
	DeSandre-Robinson et al., 2017 [14]	3.54 ± 1.62	-	5.48 ± 2.75
TTA		3.2 ± 0.64	-	4.73 ± 2.14
	Kühn et al., 2011 [22]	4.5 ± -	-	4.3 ± -
MMT	Valiño-Cultelli et al., 2022 [23]	3.31 ± 1.48	3.88 ± 1.74	4.46 ± 1.83

## Data Availability

The original contributions presented in this study are included in the article. Further inquiries can be directed to the corresponding author.
